# Cost-effectiveness of continuous glucose monitoring and intensive insulin therapy for type 1 diabetes

**DOI:** 10.1186/1478-7547-9-13

**Published:** 2011-09-14

**Authors:** R Brett McQueen, Samuel L Ellis, Jonathan D Campbell, Kavita V Nair, Patrick W Sullivan

**Affiliations:** 1 Pharmaceutical Outcomes Research Program, School of Pharmacy, University of Colorado Denver, Aurora, Colorado, USA; 2 Department of Clinical Pharmacy, School of Pharmacy, University of Colorado Denver, Denver, Aurora, Colorado, USA; 3 Department of Pharmacy Practice, Regis University, Denver, Colorado, USA

**Keywords:** Cost-effectiveness analysis, Continuous Glucose Monitoring, Type 1 diabetes, Cost-utility analysis, Self-Monitoring of Blood Glucose

## Abstract

**Background:**

Our objective was to determine the cost-effectiveness of Continuous Glucose Monitoring (CGM) technology with intensive insulin therapy compared to self-monitoring of blood glucose (SMBG) in adults with type 1 diabetes in the United States.

**Methods:**

A Markov cohort analysis was used to model the long-term disease progression of 12 different diabetes disease states, using a cycle length of 1 year with a 33-year time horizon. The analysis uses a societal perspective to model a population with a 20-year history of diabetes with mean age of 40. Costs are expressed in $US 2007, effectiveness in quality-adjusted life years (QALYs). Parameter estimates and their ranges were derived from the literature. Utility estimates were drawn from the EQ-5D catalogue. Probabilities were derived from the Diabetes Control and Complications Trial (DCCT), the United Kingdom Prospective Diabetes Study (UKPDS), and the Wisconsin Epidemiologic Study of Diabetic Retinopathy. Costs and QALYs were discounted at 3% per year. Univariate and Multivariate probabilistic sensitivity analyses were conducted using 10,000 Monte Carlo simulations.

**Results:**

Compared to SMBG, use of CGM with intensive insulin treatment resulted in an expected improvement in effectiveness of 0.52 QALYs, and an expected increase in cost of $23,552, resulting in an ICER of approximately $45,033/QALY. For a willingness-to-pay (WTP) of $100,000/QALY, CGM with intensive insulin therapy was cost-effective in 70% of the Monte Carlo simulations.

**Conclusions:**

CGM with intensive insulin therapy appears to be cost-effective relative to SMBG and other societal health interventions.

## Background

Diabetes mellitus and its complications continue to be a growing burden on the United States health care system. The American Diabetes Association (ADA) estimates that as of 2007, the prevalence of type 1 and 2 diabetes is over 24 million, growing at 1 million people diagnosed with diabetes per year since 2002 [1]. The ADA estimated an annual cost in 2007 of $174 billion due to diabetes, $116 billion of that due to direct medical costs of diabetes and chronic conditions related to diabetes [1]. There is an obvious need for reductions in costs related to diabetes while improving management of the disease, thus increasing the quality of life of persons with diabetes.

Clinical evidence shows that improvements in hemoglobin A1c levels (i.e., < 7% recommended by the ADA [1]) can reduce or delay complications related to both type 1 and 2 diabetes [2-4]. Diabetes complications include microvascular (i.e., retinopathy, nephropathy, neuropathy), macrovascular (i.e., coronary heart disease, cerebrovascular disease, peripheral artery disease), and short - term severe hypoglycemic complications [5]. Minimal reductions in A1c levels have been documented in long - term and short - term studies to reduce complications that can result in significant cost savings [6,7]. To assess glycemic control the ADA has recommendations for both glucose monitoring and A1c target levels [5]. For persons with type 1 diabetes, intensive insulin therapy (e.g., injections, pump therapy) is needed, along with self-monitoring of blood glucose (SMBG) often multiple times per day [5]. While SMBG with intensive insulin therapy has been shown to be important for managing glucose levels [2,7-9], recent evidence has shown that continuous glucose monitoring (CGM) with intensive insulin therapy reduces overall A1c levels further, while holding hypoglycemic episodes constant [10-12]. In addition, recent evidence from a clinical trial population has examined the cost-effectiveness of CGM. The authors found that CGM was cost-effective (< $100,000/QALY) for type 1 diabetes meeting their clinical trial inclusion/exclusion criteria [13]. Given the increasing evidence of the clinical and economic benefit of CGM in clinical trial populations, it is important to assess whether broadening its use to a wider U.S. population would be cost-effective.

The objective of this analysis is to assess the cost-effectiveness of CGM with intensive insulin therapy relative to standard care (i.e., SMBG with intensive insulin therapy) in a general U.S. population of individuals with type 1 diabetes.

## Methods

### Markov Cohort Simulation Model

**Figure 1:**
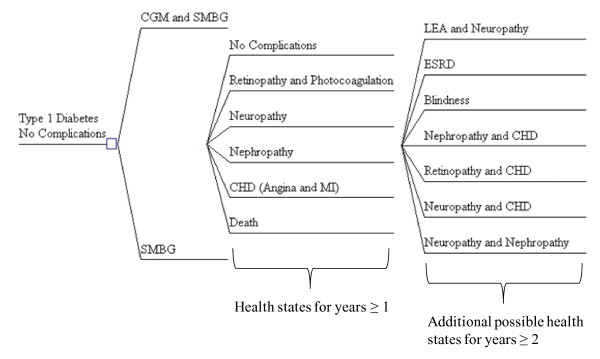
**Conceptual Markov model in decision tree format**. Both arms include self-monitoring of blood glucose (SMBG), but the technology arm includes the addition of continuous glucose monitoring (CGM). Health states are the same for both arms.

A population level Markov cohort simulation was employed to model the long-term disease progression of patients with type 1 diabetes. Long-term (i.e., micro and macrovascular) events for each arm were modeled via reductions in A1c levels. The baseline characteristics of this population cohort reflect those of the adult population (i.e., 25 years of age and older) in the Tamborlane et al. study on CGM [10]. All subjects were type 1 diabetes patients, with approximately 20 years since diagnosis, a mean age of 40 years, and a mean A1c level of 7.6% (+ or - 0.5%). A cycle length of one year was used for the Markov analysis, with a time horizon of 33 years, assuming a life expectancy of 73 years. The Markov model is represented in a decision analysis format (Figure 1), using TreeAge Pro 2009 (TreeAge Software, Williamstown, MA, USA). Continuous glucose monitoring with self-monitoring of blood glucose is compared to self-monitoring of blood glucose alone. All costs are in 2007 US dollars, and a discount rate of 3% was used for costs and QALYs.

There are many widely published and validated models, such as the CORE Diabetes Model, that project long-term diabetes outcomes [14,15]. However, we built a model targeted specifically towards the clinical benefit of CGM technology in a population with characteristics similar to the Tamborlane et al. adult type 1 diabetes study population [10]. In particular, Tamborlane et al. found a mean reduction in A1c of 0.5% over the trial time period for the adult patients using CGM technology [10]. The 0.5% reduction in A1c was used for the derivation of the four CGM risk reduction parameters in our model (Table 1). The level of detail for the calculation of input parameters in our model was not available in published CORE Diabetes Model studies. We used inputs and assumptions from the model built by the C.D.C. Cost-Effectiveness Group [16,17], other literature sources [18,19], and the expertise offered by our research team. The C.D.C. Cost-Effectiveness Group used similar modeling inputs and assumptions as were used in the CORE Diabetes Model (i.e., inputs derived from the Diabetes Control and Complications Trial (DCCT), the United Kingdom Prospective Diabetes Study (UKPDS), and other literature sources) [14-17]. Therefore, the model we built was based on similar inputs and assumptions used to develop the CORE Diabetes Model, but tailored to serve the needs of our analysis. For more information on model inputs and assumptions please see Additional File 1.

**Table 1:** Parameters for Type 1 Diabetes Markov Model

Transition Probabilities [Annual cycle length]^a^	Mean	2.5%^b^	97.50%	Reference
Retinopathy to blindness	0.101	0.057	0.156	Hoerger et al. [16,17]
Diabetes with no complications to CHD	0.031	0.018	0.048	Hoerger et al. [16,17]
Subsequent LEA	0.110	0.062	0.169	Hoerger et al. [16,17]
Diabetes with no complications to nephropathy	0.072	0.041	0.112	Klein et al. [18]
Nephropathy to CHD	0.022	0.013	0.034	Klein et al. [18]
Nephropathy to ESRD	0.072	0.041	0.109	Hoerger et al. [16,17]
Diabetes with no complications to neuropathy	0.035	0.020	0.055	Klein et al. [18]
Neuropathy to CHD	0.029	0.016	0.044	Hoerger et al. [16,17]
Neuropathy to LEA	0.131	0.074	0.200	Hoerger et al. [16,17]
Neuropathy to nephropathy	0.097	0.055	0.149	Wu et al. [19]
Diabetes with no complications to retinopathy	0.011	0.006	0.017	Hoerger et al. [16,17]
Retinopathy to CHD	0.028	0.016	0.043	Klein et al. [18]

**Cost Parameters [Annual or initial costs represented in 2007 US$]^c^**				

Blindness and retinopathy	9,912	7,251	12,945	ADA [1]
CGM technology	4,189	3,062	5,492	CGM website [24]
Initial cost of CGM technology	4,809	3,499	6,321	CGM website [24]
CHD	35,271	25,820	46,433	ADA [1]
Diabetes with no complications	6,705	4,879	8,788	ADA [1]
ESRD	36,370	26,377	47,708	ADA [1]
LEA	50,150	36,541	65,798	ADA [1]
Nephropathy	20,161	14,614	26,643	ADA [1]
Neuropathy	25,075	18,226	33,004	ADA [1]
Retinopathy	4,956	3,578	6,489	ADA [1]

**Utility Parameters [Annual cycle length]^a^**				

Blindness	0.569	0.531	0.607	Sullivan et al. [22] ICD-9 250
CHD	0.552	0.513	0.591	Sullivan et al. [22] ICD-9 250, 593
ESRD	0.521	0.485	0.558	Sullivan et al. [22] ICD-9 250, 355
LEA	0.572	0.538	0.604	Sullivan et al. [22] ICD-9 250, 362
Nephropathy	0.575	0.545	0.606	Sullivan et al. [22] ICD-9 250, 355, 593
Nephropathy and CHD	0.516	0.465	0.567	Sullivan et al. [22] ICD-9 250, 593, 410, 413
Neuropathy	0.603	0.573	0.632	Sullivan et al. [22] ICD-9 250, 355, 410, 413
Neuropathy and CHD	0.544	0.495	0.593	Sullivan et al. [22] ICD-9 250, 362, 410, 413
Neuropathy and nephropathy	0.557	0.520	0.595	Sullivan et al. [22] ICD-9 250, 410, 413
Diabetes with no complications	0.757	0.747	0.767	Sullivan et al. [22] ICD-9 250, 593, 586
Retinopathy	0.612	0.581	0.643	Sullivan et al. [22] ICD-9 250, 355, 354
Retinopathy and CHD	0.553	0.503	0.605	Sullivan et al. [22] ICD-9 250, 362, 369
Disutility of age	-0.0003			Sullivan et al. [22]

**Other Parameters^d^**				

CGM risk reduction for CHD	0.050	0.013	0.107	DCCT [20]
CGM risk reduction for nephropathy	0.270	0.006	0.768	DCCT [20]
CGM risk reduction for neuropathy	0.188	0.004	0.593	DCCT [20]
CGM risk reduction for retinopathy	0.306	0.075	0.618	Selvin et al. [21]
Start age	40			Assumption
Years since diagnosis	20			Assumption
Discount rate	0.03			Assumption

a Beta distribution assumed

b Credible range of values from the 2.5th and 97.5th percentiles of the 10,000 second order Monte Carlo simulations

c Gamma distribution assumed for all cost parameters

d Beta distribution assumed for all risk reduction parameters; start age, years since diagnosis, and discount rate were not varied

In this model, all members of the population start with no complications. After this, the population can transition to one of six health states including retinopathy, nephropathy, neuropathy, Coronary Heart Disease (CHD), continue with diabetes and no complications, or death. From the five disease states, the population may then enter an additional seven disease states: nephropathy and CHD, neuropathy and CHD, retinopathy and CHD, neuropathy and nephropathy, blindness, end stage renal disease, lower extremity amputation and neuropathy, or death (transition probabilities shown in Table 1). Patients can develop a maximum of four concomitant chronic comorbidities in the Markov model.

### Input Parameters

As delineated in Table 1, transition probabilities are drawn from the best available estimates from the literature [16-19]. Based on evidence from Klein et al. [18], the transition probabilities of going from nephropathy to CHD (0.022), neuropathy to CHD (0.029), and retinopathy to CHD (0.028) are equal to the estimates of going from CHD back to the respective microvascular disease states. The transition probability from neuropathy to nephropathy (0.097) is conditional and drawn directly from Wu et al [19]. When the population enters concomitant disease states such as neuropathy and nephropathy for example, they are limited to that state for the rest of the cycle. The transition back into each concomitant disease state is the complimentary probability based on mortality rates (available in Additional File 1).

The probability estimates just described show the progression of diabetes for those with an average A1c level of around 8%. CGM has been shown to reduce A1c levels by 0.5% in adult patients [10]. CGM exhibited its relative risk reduction for development of chronic comorbidity as a result of its reduction in A1c levels. Risk reduction parameters were drawn from two sources: the DCCT [20] for microvascular complications, and a meta - analysis relating to macrovascular complications by Selvin et al [21].

Utility values for each disease state were taken from the EQ-5D catalogue by Sullivan et al (Table 1) [22]. Each disease state begins with the unadjusted mean EQ-5D score from the population in MEPS 2000-2002 with diabetes mellitus, adjusted to reflect a mean age of 40 years. The utility calculation for each disease state also includes deductions for age by cycle length, and discounting by 3% [23]. There are a total of 12 different utilities for each disease state. Incremental effectiveness is expressed in quality-adjusted life years (QALYs) gained.

Costs were derived from evidence published by the ADA [1]. The annual mean cost of diabetes represents the per capita expenditures for people with diabetes at all age groups for hospital inpatient visits, nursing/residential facility visits, physician's office visits, emergency department (ED) trips, hospital outpatient visits, home health care, hospice care, podiatry care, insulin, diabetic supplies, oral agents, retail prescriptions, other supplies, and patient time [1]. Lost wages served as a proxy for patient time. The ADA estimates that people with diabetes experience an additional 2.5 days absent compared to those without diabetes [1]. The authors also estimated that the same population with diabetes on average earns $250 a day. They also estimate that the population aged 64 or less has approximately $625 of patient time per year for annual treatment of diabetes [1]. The assumption for the population over 64 is one day of lost wages ($250). Other costs in the model include marginal annual costs for each disease state, such as blindness, end stage renal disease, lower extremity amputation and neuropathy, retinopathy, neuropathy, nephropathy, and CHD, along with the concomitant disease states. The marginal costs for each disease state were calculated using average length of stay in an inpatient hospital setting and the cost per medical event, estimated from the ADA [1]. Costs per health state are delineated in Table 1. The concomitant disease states were estimated by summing the marginal cost for each disease state, with the exception of blindness, lower extremity amputation, and end stage renal disease (i.e., neuropathy and CHD, nephropathy and CHD, retinopathy and CHD, neuropathy and nephropathy, where each were calculated separately). While the summation assumption for marginal costs of each combination of disease states may overestimate the costs associated with having those disease states, the ADA does note their cost estimates are an underestimate of the societal cost attributable to diabetes [1]. CGM costs were estimated from a diabetes technology and treatment purchasing website [24]. Annual and initial costs are an average based on 3 systems, the Guardian Real - Time, Dexcom seven, and MiniMed Paradigm Real - Time system. The initial cost of CGM ($4,809) consists of the monitor, transmitter, two hours of patient time for education, and sensors for the first year. The annual costs ($4,189) thereafter include additional sensors per year, two hours of patient time for maintenance, and additional transmitters and batteries for the year. The initial CGM cost estimate is included in the zero cycle of the Markov model node CGM. The annual cost of CGM is then included in all disease states including no complications after cycle zero.

The all cause mortality rate was based on an average of all race categories (Non-Hispanic white, African-American, Hispanic, Native American, and Asian), and gender, from the C.D.C. Cost-Effectiveness group [16]. Increased mortality risks were drawn from the Early Treatment Diabetic Retinopathy Study (ETDRS) by Cusick et al [25]. The tables for each mortality rate (neuropathy, nephropathy, CHD, LEA, and ESRD, and each concomitant disease state) are available in Additional File 1.

### Sensitivity Analysis

Probabilistic sensitivity analysis was performed using Monte Carlo simulation to evaluate the multivariate uncertainty in the model. The input parameters were varied simultaneously over specified ranges. Various probability distributions were chosen based on assumptions for each input parameter. The beta distribution was specified for the probability, utility, and risk reduction parameters. The Gamma distribution was specified for the cost parameters. The Monte Carlo simulation drew values for each input parameter and calculated expected cost and effectiveness for each arm of the model. This process was repeated 10,000 times to give a range of all expected cost and effectiveness values. Additionally, univariate sensitivity analysis was conducted to identify variables that had the largest impact on the model results. For the univariate sensitivity analysis we varied all parameters shown in Table 1 by +/- 15%. The parameters that had the largest impact on the model results are presented in a tornado diagram. The top ten variables from the tornado diagram were individually varied by 50% to estimate the effect on the model results.

## Results

### Base - Case Analysis

The results for the base-case analysis are shown in Table 2. The mean total lifetime costs for SMBG were $470,583. The mean total lifetime costs for SMBG and CGM technology totaled $494,135, resulting in an incremental cost of $23,552. Lifetime effectiveness for SMBG was 10.289 QALYs. Lifetime effectiveness for SMBG with the addition of CGM technology was 10.812 QALYs, resulting in an incremental effectiveness of 0.523 QALYs. The incremental cost-effectiveness ratio (ICER) was $45,033 per QALY for CGM technology. Mortality was not directly reduced by CGM; it simply reduced the probability of entering disease states, thereby delaying the increased mortality from complications.

**Table 2:** Expected Cost and Effectiveness of Continuous Glucose Monitoring (CGM) and Self-Monitoring of Blood Glucose (SMBG)

Strategy	Expected Cost in 2007 $US (range)*	Expected Effectiveness QALYs (range)*	Incremental cost-effectiveness ratio (ICER)
SMBG	470,583 (397,782 - 550,598)	10.289 (9.615 - 10.957)	
CGM and SMBG	494,135 (420,381 - 571,631)	10.812 (9.894 - 11.887)	US $45,033/QALY

*95% credible ranges based on the results from the 10,000 Monte Carlo simulations

### Sensitivity Analysis

**Figure 2:**
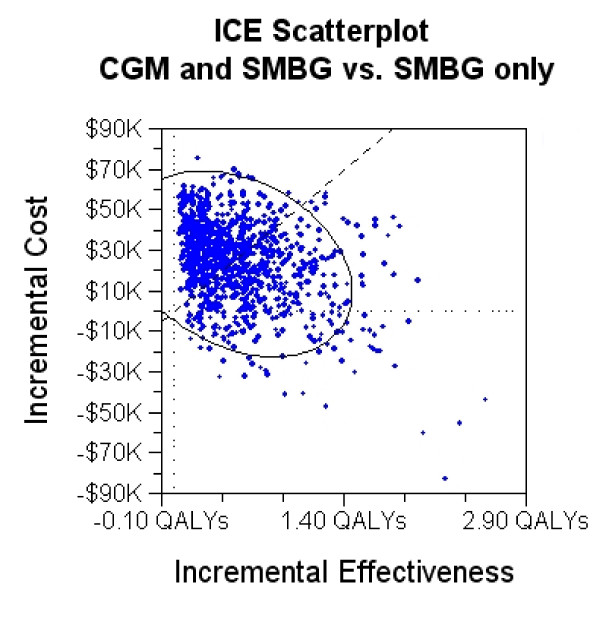
**Incremental cost-effectiveness scatter plot: CGM and SMBG vs. SMBG only**. Incremental cost-effectiveness scatter plot of continuous glucose monitoring (CGM) and self-monitoring of blood glucose (SMBG) vs. SMBG only. The diagonal dashed line represents US$50,000 per quality-adjusted life year. Each point represents one Monte Carlo simulation.

Results of the probabilistic sensitivity analysis are shown in Table 2 and Figure 2. The ranges given in Table 2 are 95% credible ranges for the expected cost and effectiveness. Figure 2 is a scatter plot of incremental cost-effectiveness pairs for the use of CGM with SMBG vs. SMBG only. The dashed diagonal line represents US$50,000 per QALY. Each dot represents one simulation. The ICER estimates in the southeast quadrant make up 10.66% of the simulations, and indicate that CGM is less costly and more effective, dominating SMBG. The rest of the simulations lie in the northeast quadrant with 36.96% below US$50,000/QALY. Results show that 48% of the observations are cost-effective for a willingness-to-pay of US$50,000 per QALY and 70% for a WTP of $100,000/QALY.

**Figure 3:**
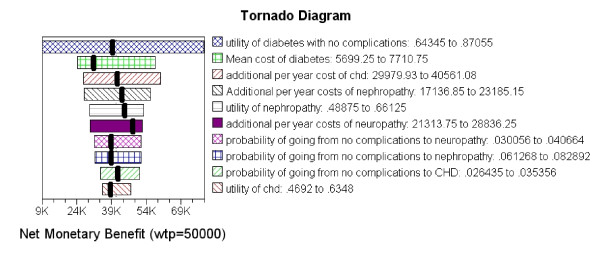
**Tornado diagram of the variables that have the largest impact on the model results**. The ten variables with the largest impact on the model results (each while holding all other variables constant) are listed in descending order. Utility of diabetes with no complications had the largest impact on the model results.

The univariate sensitivity analysis results are shown in Figure 3 as a tornado diagram, expressed in terms of net monetary benefit. Net monetary benefit is calculated by taking the difference in effectiveness and multiplying by society's willingness-to-pay, less the difference in costs. After identifying the ten variables with the largest impact on the model results, each was varied individually by 50%. The utility of diabetes with no complications, the annual cost of CHD, and the probability of going from diabetes with no complications to the CHD disease state, had the largest impact on the model results. The utility of diabetes with no complications was decreased by 50%, and the corresponding incremental effectiveness dramatically decreased, resulting in an ICER over US$300,000/QALY. When the utility of diabetes with no complications was increased by 50%, incremental effectiveness increased, decreasing the ICER to approximately US$30,000/QALY. The annual cost of CHD also had a large impact on the model results, and when decreased by 50%, the ICER was US$86,000/QALY. When the annual cost of CHD was increased by 50% the ICER was US$12,000/QALY. The probability of going from diabetes with no complications to the CHD disease state was decreased by 50%, estimating an ICER of approximately US$66,000/QALY. When the probability of entering the CHD disease state was increased by 50% the ICER was US$32,000/QALY. The other variables listed in the tornado diagram were also varied by 50%, but offered no meaningful impact on the model results (within the range of US$40,000/QALY to US$60,000/QALY).

## Discussion

CGM may be an important clinical technology for managing diabetes. The objective of this analysis was to determine the cost-effectiveness of CGM at a population level. The current model estimated the progression of chronic disease in a population with type 1 diabetes. CGM reduced the progression of chronic disease and mortality relative to SMBG alone. The base case analysis resulted in an ICER of US$45,033/QALY. Results from the probabilistic sensitivity analysis indicate 48% of the Monte Carlo simulations were under US$50,000/QALY, while 70% were under US$100,000/QALY. These results suggest that CGM is cost-effective compared with SMBG and other societal health interventions.

There are limitations to this analysis. The probability values are from different sample populations. The probabilities are constant with each cycle, indicating no increase in the risk of complications due to diabetes over time. Given that the baseline probabilities reflect a population of very ill patients with type 1 diabetes, the assumption may still be valid, particularly for the cohort averages (which this analysis models). The cumulative incidence of CHD (Angina and myocardial infarction) from Klein et al. was not significantly associated with A1c levels [18]. In other words, increasing levels of A1c were not significantly associated with the incidence of CHD. Nevertheless, we assumed an A1c level of 8% when deriving the transition probability into each state involving CHD. This model also did not explicitly model hypoglycemic events. This is a significant drawback considering many type 1 diabetes patients specifically purchase a continuous monitor for reductions in hypoglycemic events. However, the data on the ability of CGM to reduce hypoglycemic events is not conclusive and thus it was not included in the model. As the evidence becomes clearer, future models should examine its impact. This model also did not explicitly model hypertension control, which is known to impact the development of diabetes complications. Hypertension control was also omitted from the structural model because it was not clear from current evidence that CGM would differentially affect hypertension control.

The previous cost-effectiveness analysis by Huang et al. found an immediate quality-of life-benefit for the patients using CGM [13]. Although considerable uncertainty was present, long-term projections indicated an average gain in QALYs of 0.60 and an ICER of less than $100,000/QALY. The cost-effectiveness analysis by Huang et al. provides important information about CGM in a restricted clinical trial population. This analysis differs from that of Huang et al. in several significant ways. To begin, our analysis reflects the societal perspective. The cohort modeled was chosen to reflect a general population of individuals with type 1 diabetes and was not restricted to a specific clinical trial population. The utilities in our study were taken from the EQ-5D catalogue, which were derived from a nationally representative population and the underlying EQ-5D tariffs were from a U.S. community population. Our model also includes explicit concomitant disease states, which may be a better representation of the clinical pathway associated with diabetes.

## Conclusions

While the model has many limitations, it provides a valid picture of diabetes disease progression and the effect of lowering A1c levels in a representative general population of individuals with type 1 diabetes. This analysis shows that CGM may be a cost-effective means of lowering disease progression and complications via its impact on A1c levels. Previous studies have documented the beneficial clinical effects of CGM in this population. Our study adds to this body of evidence by suggesting that CGM may also provide a cost-effective means of lowering A1c in a general population. As long as the evidence continues to suggest that use of CGM helps to lower A1c levels, it is important for individuals with type 1 diabetes to have affordable access to and education about this technology. This study suggests that for individuals with type 1 diabetes and A1c above 8%, CGM and SMBG with intensive insulin therapy is a cost-effective alternative to SMBG alone with intensive insulin therapy.

## List of Abbreviations

ADA: stands for American Diabetes Association; CGM: is Continuous Glucose Monitoring; CHD: is Coronary Heart Disease; DCCT: is the Diabetes Control and Complications Trial; ESRD: is End-Stage Renal Disease; ETDRS: is the Early Treatment Diabetic Retinopathy Study; LEA: is Lower Extremity Amputation; QALYs: are quality-adjusted life years; SMBG: is Self-Monitoring of Blood Glucose; UKPDS: is the United Kingdom Prospective Diabetes Study; and WTP: is willingness-to-pay.

## Competing interests

The authors declare that they have no competing interests. The authors designed, conducted, and reported this research without funding or any external assistance.

## Authors' contributions

RBM drafted the manuscript. All authors participated in the design of the Markov model. SLE reviewed and revised the clinical plausibility of the model. PWS reviewed and revised the Markov model assumptions, and interpretation of the model results. JDC and KVN revised Figure 1 and wrote portions of the revised Methods section. All authors read, revised, and approved the final manuscript.

## Supplementary Material

Additional file 1 **Appendix for Cost-Effectiveness of Continuous Glucose Monitoring and Intensive Insulin Therapy for Type 1 Diabetes**. This technical appendix provides further information regarding the assumptions and calculations of the Markov Cohort simulation. Appendix Table 1A shows the assumed distributional properties and moments of the respective distributions. Appendix Table 2A and 2B show information on mortality rates. Appendix Table 3 and 4 show more information related to Diabetes costs, and costs related to CGM technology. Click here for file
