# Supplementary material for: Cost-effectiveness of continuous glucose monitoring and intensive insulin therapy for type 1 diabetes
Source: Cost Eff Resour Alloc. 2011 Sep 14;9:13. doi: 10.1186/1478-7547-9-13 (PMC3180394; doi:10.1186/1478-7547-9-13)
Supplement: Additional file 1 — Appendix for Cost-Effectiveness of Continuous Glucose Monitoring and Intensive Insulin Therapy for Type 1 Diabetes. This technical appendix provides further information regarding the assumptions and calculations of the Markov Cohort simulation. Appendix Table 1A shows the assumed distributional properties and moments of the respective distributions. Appendix Table 2A and 2B show information on mortality rates. Appendix Table 3 and 4 show more information related to Diabetes costs, and costs related to CGM technology. [file 1478-7547-9-13-S1.PDF]

Appendix for Cost-Effectiveness of Continuous Glucose Monitoring and Intensive Insulin Therapy for Type 1 Diabetes

Robert B. McQueen[1], Samuel L. Ellis[2], Jonathan D. Campbell[1], Kavita V. Nair[1], Patrick W. Sullivan[3]

[1]Pharmaceutical Outcomes Research Program, School of Pharmacy, University of Colorado Denver (AMC), Aurora, Colorado, USA

[2]Department of Clinical Pharmacy, School of Pharmacy, University of Colorado Denver (AMC), Denver, Aurora, Colorado, USA

[3]Department of Pharmacy Practice, Regis University, Denver, Colorado, USA

This technical appendix provides further information regarding the assumptions and calculations of the Markov Cohort simulation. Appendix Table 1A shows the assumed distributional properties and moments of the respective distributions. Appendix Table 2A and 2B show information on mortality rates. Appendix Table 3 and 4 show more information related to Diabetes costs, and costs related to CGM technology.

Appendix Table 1A: Parameters for Type 1 Diabetes Markov Model and Distributional Properties

**Transition Probabilities [Annual cycle length]**	**Mean**	**SE**	**Minimum**	**2.5%**	**Median**	**97.50%**	**Maximum**	**Reference**
Retinopathy to Blindness	0.101	0.025	0.032	0.057	0.099	0.156	0.230	Hoerger et al. [16, 17]
Diabetes with no complications to CHD	0.031	0.008	0.011	0.018	0.031	0.048	0.070	Hoerger et al. [16, 17]
Subsequent LEA	0.110	0.027	0.032	0.062	0.108	0.169	0.239	Hoerger et al. [16, 17]
Diabetes with no complications to Nephropathy	0.072	0.018	0.022	0.041	0.071	0.112	0.161	Klein et al. [18]
Nephropathy to CHD	0.022	0.006	0.007	0.013	0.022	0.034	0.050	Klein et al. [18]
Nephropathy to ESRD	0.072	0.018	0.021	0.041	0.070	0.109	0.186	Hoerger et al. [16, 17]
Diabetes with no complications to neuropathy	0.035	0.009	0.011	0.020	0.035	0.055	0.077	Klein et al. [18]
Neuropathy to CHD	0.029	0.007	0.009	0.016	0.028	0.044	0.064	Hoerger et al. [16, 17]
Neuropathy to LEA	0.131	0.033	0.036	0.074	0.128	0.200	0.274	Hoerger et al. [16, 17]
Neuropathy to Nephropathy	0.097	0.024	0.032	0.055	0.095	0.149	0.210	Wu et al. [19]
Diabetes with no complications to retinopathy	0.011	0.003	0.003	0.006	0.011	0.017	0.026	Hoerger et al. [16, 17]
Retinopathy to CHD	0.028	0.007	0.008	0.016	0.027	0.043	0.062	Klein et al. [18]
**Cost Parameters [Annual or initial costs represented in 2007 US$]**								
Blindness and Retinopathy	9912	1462	5720	7251	9838	12945	16947	ADA[1]
CGM Technology	4189	623	2249	3062	4144	5492	6752	CGM website [24]
Initial Cost of CGM Technology	4809	727	2370	3499	4768	6321	8341	CGM website [24]
CHD	35271	5281	16957	25820	35111	46433	61320	ADA[1]
Diabetes with no complications	6705	1001	3701	4879	6659	8788	11390	ADA[1]
ESRD	36370	5444	18884	26377	36065	47708	59888	ADA[1]
LEA	50150	7563	26180	36541	49728	65798	81678	ADA[1]
Nephropathy	20161	3042	10520	14614	20001	26643	33706	ADA[1]
Neuropathy	25075	3741	13110	18226	24883	33004	41126	ADA[1]
Retinopathy	4956	753	2681	3578	4921	6489	8269	ADA[1]
**Utility Parameters [Annual cycle length]**								
Blindness	0.569	0.019	0.495	0.531	0.569	0.607	0.638	Sullivan et al. [22] ICD-9 250
CHD	0.552	0.020	0.481	0.513	0.552	0.591	0.629	Sullivan et al. [22] ICD-9 250, 593
ESRD	0.521	0.019	0.446	0.485	0.521	0.558	0.594	Sullivan et al. [22] ICD-9 250, 355
LEA	0.572	0.017	0.506	0.538	0.572	0.604	0.667	Sullivan et al. [22] ICD-9 250, 362
Nephropathy	0.575	0.016	0.505	0.545	0.575	0.606	0.632	Sullivan et al. [22] ICD-9 250, 355, 593
Nephropathy and CHD	0.516	0.026	0.413	0.465	0.517	0.567	0.612	Sullivan et al. [22] ICD-9 250, 593, 410, 413
Neuropathy	0.603	0.015	0.543	0.573	0.603	0.632	0.661	Sullivan et al. [22] ICD-9 250, 355, 410, 413
Neuropathy and CHD	0.544	0.025	0.448	0.495	0.544	0.593	0.638	Sullivan et al. [22] ICD-9 250, 362, 410, 413

Neuropathy and Nephropathy	0.557	0.019	0.474	0.520	0.557	0.595	0.624	Sullivan et al. [22] ICD-9 250, 410, 413
Diabetes with no complications	0.757	0.005	0.736	0.747	0.757	0.767	0.777	Sullivan et al. [22] ICD-9 250, 593, 586
Retinopathy	0.612	0.016	0.548	0.581	0.612	0.643	0.669	Sullivan et al. [22] ICD-9 250, 355, 354
Retinopathy and CHD	0.553	0.026	0.461	0.503	0.553	0.605	0.643	Sullivan et al. [22] ICD-9 250, 362, 369
Disutility of age	-0.0003							Sullivan et al. [22]
**Other Parameters**								
CGM risk reduction for CHD	0.050	0.025	0.002	0.013	0.046	0.107	0.211	DCCT [20]
CGM risk reduction for nephropathy	0.270	0.214	0.000	0.006	0.218	0.768	0.991	DCCT [20]
CGM risk reduction for neuropathy	0.188	0.163	0.000	0.004	0.143	0.593	0.888	DCCT [20]
CGM risk reduction for retinopathy	0.306	0.144	0.009	0.075	0.293	0.618	0.839	Selvin et al. [21]
start age	40							Assumption
years since diagnosis	20							Assumption
discount rate	0.03							Assumption

Appendix Table 1B: Approximation of Distributions for Input Parameters	
**Transition Probabilities (Annual cycle length)**	**Approximation of Distribution**
Retinopathy to Blindness	Beta, Real-numbered parameters, alpha = ((.101^2)*(1-.101)/(.02525^2)), beta = (.101*(1-.101)/(.02525^2))-((.101^2)*(1-.101)/(.02525^2)); Expected value: 0.101
Diabetes with no complications to CHD	Beta, Real-numbered parameters, alpha = ((.0311^2)*(1-.0311)/(.00775^2)), beta = (.0311*(1-.0311)/(.00775^2))-((.0311^2)*(1-.0311)/(.00775^2)); Expected value: 0.0311
Subsequent LEA	Beta, Real-numbered parameters, alpha = ((.11^2)*(1-.11)/(.0275^2)), beta = (.11*(1-.11)/(.0275^2))-((.11^2)*(1-.11)/(.0275^2)); Expected value: 0.11
Diabetes with no complications to Nephropathy	Beta, Real-numbered parameters, alpha = ((.07208^2)*(1-.07208)/(.01802^2)), beta = (.07208*(1-.07208)/(.01802^2))-((.07208^2)*(1-.07208)/(.01802^2)); Expected value: 0.07208
Nephropathy to CHD	Beta, Real-numbered parameters, alpha = ((.02225^2)*(1-.02225)/(.0055625^2)), beta = (.02225*(1-.02225)/(.0055625^2))-((.02225^2)*(1-.02225)/(.0055625^2)); Expected value: 0.02225
Nephropathy to ESRD	Beta, Real-numbered parameters, alpha = ((.0713^2)*(1-.0713)/(.017825^2)), beta = (.0713*(1-.0713)/(.017825^2))-((.0713^2)*(1-.0713)/(.017825^2)); Expected value: 0.0713
Diabetes with no complications to neuropathy	Beta, Real-numbered parameters, alpha = ((.03536^2)*(1-.03536)/(.00884^2)), beta = (.03536*(1-.03536)/(.00884^2))-((.03536^2)*(1-.03536)/(.00884^2)); Expected value: 0.03536
Neuropathy to CHD	Beta, Real-numbered parameters, alpha = ((.0286^2)*(1-.0286)/(.00715^2)), beta = (.0286*(1-.0286)/(.00715^2))-((.0286^2)*(1-.0286)/(.00715^2)); Expected value: 0.0286
Neuropathy to LEA	Beta, Real-numbered parameters, alpha = ((.1306^2)*(1-.1306)/(.03265^2)), beta = (.1306*(1-.1306)/(.03265^2))-((.1306^2)*(1-.1306)/(.03265^2)); Expected value: 0.1306
Neuropathy to Nephropathy	Beta, Real-numbered parameters, alpha = ((.097^2)*(1-.097)/(.02425^2)), beta = (.097*(1-.097)/(.02425^2))-((.097^2)*(1-.097)/(.02425^2)); Expected value: 0.097
Diabetes with no complications to retinopathy	Beta, Real-numbered parameters, alpha = ((.01094^2)*(1-.01094)/(.002735^2)), beta = (.01094*(1-.01094)/(.002735^2))-((.01094^2)*(1-.01094)/(.002735^2)); Expected value: 0.01094
Retinopathy to CHD	Beta, Real-numbered parameters, alpha = ((.0277^2)*(1-.0277)/(.006925^2)), beta = (.0277*(1-.0277)/(.006925^2))-((.0277^2)*(1-.0277)/(.006925^2)); Expected value: 0.0277
**Cost Parameters (Annual or initial costs represented in 2007 US$)**	
Blindness and Retinopathy	Gamma, alpha = (9912^2)/(1487^2), lambda = 9912/(1487^2); Expected value: 9912
CGM Technology	Gamma, alpha = (4189^2)/(628.35^2), lambda = 4189/(628.35^2); Expected value: 4189
Initial Cost of CGM Technology	Gamma, alpha = (4809^2)/(721.35^2), lambda = 4809/(721.35^2); Expected value: 4809
CHD	Gamma, alpha = (35271^2)/(5291^2), lambda = 35271/(5291^2); Expected value: 35271
Diabetes with no complications	Gamma, alpha = (6705^2)/(1006^2), lambda = 6705/(1006^2); Expected value: 6705
ESRD	Gamma, alpha = (36370^2)/(5456^2), lambda = 36370/(5456^2); Expected value: 36370
LEA	Gamma, alpha = (50150^2)/(7523^2), lambda = 50150/(7523^2); Expected value: 50150
Nephropathy	Gamma, alpha = (20161^2)/(3024^2), lambda = 20161/(3024^2); Expected value: 20161

Neuropathy	Gamma, alpha = (25075^2)/(3761^2), lambda = 25075/(3761^2); Expected value: 25075
Retinopathy	Gamma, alpha = (4956^2)/(743^2), lambda = 4956/(743^2); Expected value: 4956
**Utility Parameters (Annual cycle length)**	
Blindness	Beta, Real-numbered parameters, alpha = ((.569^2)*(1-.569)/(.019253^2)), beta = (.569*(1-.569)/(.019253^2))-((.569^2)*(1-.569)/(.019253^2)); Expected value: 0.569
CHD	Beta, Real-numbered parameters, alpha = ((.552^2)*(1-.552)/(.020125^2)), beta = (.552*(1-.552)/(.020125^2))-((.552^2)*(1-.552)/(.020125^2)); Expected value: 0.552
ESRD	Beta, Real-numbered parameters, alpha = ((.521^2)*(1-.521)/(.018712^2)), beta = (.521*(1-.521)/(.018712^2))-((.521^2)*(1-.521)/(.018712^2)); Expected value: 0.521
LEA	Beta, Real-numbered parameters, alpha = ((.572^2)*(1-.572)/(.01692^2)), beta = (.572*(1-.572)/(.01692^2))-((.572^2)*(1-.572)/(.01692^2)); Expected value: 0.572
Nephropathy	Beta, Real-numbered parameters, alpha = ((.575^2)*(1-.575)/(.01585^2)), beta = (.575*(1-.575)/(.01585^2))-((.575^2)*(1-.575)/(.01585^2)); Expected value: 0.575
Nephropathy and CHD	Beta, Real-numbered parameters, alpha = ((.516^2)*(1-.516)/(.02617^2)), beta = (.516*(1-.516)/(.02617^2))-((.516^2)*(1-.516)/(.02617^2)); Expected value: 0.516
Neuropathy	Beta, Real-numbered parameters, alpha = ((.603^2)*(1-.603)/(.014826^2)), beta = (.603*(1-.603)/(.014826^2))-((.603^2)*(1-.603)/(.014826^2)); Expected value: 0.603
Neuropathy and CHD	Beta, Real-numbered parameters, alpha = ((.544^2)*(1-.544)/(.025058^2)), beta = (.544*(1-.544)/(.025058^2))-((.544^2)*(1-.544)/(.025058^2)); Expected value: 0.544
Neuropathy and Nephropathy	Beta, Real-numbered parameters, alpha = ((.557^2)*(1-.557)/(.018965^2)), beta = (.557*(1-.557)/(.018965^2))-((.557^2)*(1-.557)/(.018965^2)); Expected value: 0.557
Diabetes with no complications	Beta, Real-numbered parameters, alpha = ((.757^2)*(1-.757)/(.005271^2)), beta = (.757*(1-.757)/(.005271^2))-((.757^2)*(1-.757)/(.005271^2)); Expected value: 0.757
Retinopathy	Beta, Real-numbered parameters, alpha = ((.612^2)*(1-.612)/(.01581^2)), beta = (.612*(1-.612)/(.01581^2))-((.612^2)*(1-.612)/(.01581^2)); Expected value: 0.612
Retinopathy and CHD	Beta, Real-numbered parameters, alpha = ((.553^2)*(1-.553)/(.026102^2)), beta = (.553*(1-.553)/(.026102^2))-((.553^2)*(1-.553)/(.026102^2)); Expected value: 0.553
Disutility of age	
**Other Parameters**	
CGM risk reduction for CHD	Beta, Real-numbered parameters, alpha = ((.05^2)*(1-.05)/(.025^2)), beta = (.05*(1-.05)/(.025^2))-((.05^2)*(1-.05)/(.025^2)); Expected value: 0.05
CGM risk reduction for nephropathy	Beta, Real-numbered parameters, alpha = ((.26875^2)*(1-.26875)/(.134375^2)), beta = (.26875*(1-.26875)/(.134375^2))-((.26875^2)*(1-.26875)/(.134375^2)); Expected value: 0.26875
CGM risk reduction for neuropathy	Beta, Real-numbered parameters, alpha = ((.1875^2)*(1-.1875)/(.09375^2)), beta = (.1875*(1-.1875)/(.09375^2))-((.1875^2)*(1-.1875)/(.09375^2)); Expected value: 0.1875
CGM risk reduction for retinopathy	Beta, Real-numbered parameters, alpha = ((.30625^2)*(1-.30625)/(.153125^2)), beta = (.30625*(1-.30625)/(.153125^2))-((.30625^2)*(1-.30625)/(.153125^2)); Expected value: 0.30625

Appendix Table 2A

Table 2a: Increased Mortality Rate by Disease State	
	Risk Increase
Macrovascular	1.96
Nephropathy (Severe proteinuria)	2.23
Neuropathy (Moderate)	1.51
Neuropathy (Amputation)	3.98

Appendix Table 2b: All cause mortality rate			
	Male	Female	
Age	table values	table values	Avg. Table Values
40	0.004	0.004	0.004
41	0.004	0.004	0.004
42	0.004	0.004	0.004
43	0.004	0.004	0.004
44	0.004	0.004	0.004
45	0.005	0.005	0.005
46	0.005	0.005	0.005
47	0.005	0.005	0.005
48	0.005	0.005	0.005
49	0.005	0.005	0.005
50	0.007	0.007	0.007
51	0.007	0.007	0.007
52	0.007	0.007	0.007
53	0.007	0.007	0.007
54	0.007	0.007	0.007
55	0.013	0.009	0.011
56	0.013	0.009	0.011
57	0.013	0.009	0.011
58	0.013	0.009	0.011
59	0.013	0.009	0.011
60	0.017	0.012	0.014
61	0.017	0.012	0.014
62	0.017	0.012	0.014
63	0.017	0.012	0.014
64	0.017	0.012	0.014
65	0.027	0.018	0.022
66	0.027	0.018	0.022
67	0.027	0.018	0.022
68	0.027	0.018	0.022
69	0.027	0.018	0.022
70	0.037	0.024	0.031
71	0.037	0.024	0.031
72	0.037	0.024	0.031
73	0.037	0.024	0.031

Appendix Table 3: Base costs taken from ADA: 2007 Economic costs of Diabetes (All 2007 US$)[a]

	Age Group		
	< 45	45-64	>64
**Diabetes Costs**			
Hospital Inpatient	1621	2303	5302
Nursing/residential facility	59	191	869
Physicians office	397	497	720
ED	373	173	216
Ambulance services	1	10	3
Hospital outpatient	230	164	154
Home health	0	228	564
Hospice	0	0	4
Podiatry	5	7	30
Insulin	281	195	207
Diabetic Supplies	77	107	106
Oral Agents	344	520	518
Retail Prescriptions	392	644	965
Other supplies	27	56	55
Patient time[b]	625	625	250
Total per year	4432	5720	9963

a Per person per year expenditures attributed to diabetes

b Based on 2.5 days per year on average lost due to diabetes, with an average salary of $250 per day

Appendix Table 4: CGM Utilization and Costs

Resource	Use per year	Cost per year (low-high value) 2007 US$	Assumptions
Monitor/transmitter	One time cost	800 - 1339	Initial cost, assumes no further monitor needed
Sensors	52 - 121 sensors	3120 - 4235	Based on sensor use per week, over one year
Patient time startup	2 hours	62.5	Average wage of $250 per day used, assumed extra 2 hours per year for regular maintenance and check-ups
Additional transmitters/batteries	1 new transmitter per year w/batt	250 - 649	Assumes replacement of transmitter once per year w/rechargeable pack
Total up front cost/first year		3982 - 5636	Monitor/transmitter, plus 2 hours of patient time, and sensors for the year
Total per year, after first		3432 - 4946	Sensors for the year, 2 hours of patient time, and additional transmitters/batteries for one year

References

1) American Diabetes Association: **Economic Costs of Diabetes in the U.S. in 2007**. *Diabetes Care* 2008, **31**(3): 596-615.
2) The Diabetes Control and Complications Trial Research Group: **The Effect Of Intensive Treatment Of Diabetes On The Development And Progression Of Long-Term Complications In Insulin-Dependent Diabetes Mellitus**. *New England Journal of Medicine* 1993, **329**(14): 977-986.
3) Nathan, DM, Cleary, PA, Backlund, JY, Genuth SM, Lachin JM, Orchard TJ, Raskin P, Zinman B: **Intensive Diabetes Treatment and Cardiovascular Disease in Patients with Type 1 Diabetes**. *New England Journal of Medicine* 2005, **353**(25): 2643-2653.
4) Writing Team for the Diabetes Control and Complications Trial/Epidemiology of Diabetes: **Sustained Effect of Intensive Treatment of Type 1 Diabetes Mellitus on Development and Progression of Diabetic Nephropathy: The Epidemiology of Diabetes Interventions and Complications (EDIC) Study**. *JAMA* 2003, **290**(16): 2159-2167.
5) American Diabetes Association: **Standards of Medical Care in Diabetes-2009**. *Diabetes Care* 2009, **32** Supplement(1): S13-S61.
6) Wagner EH, Sandhu N, Newton KM, McCulloch DK, Ramsey SD, Grothaus LC: **Effect of Improved Glycemic Control on Health Care Costs and Utilization**. *JAMA* 2001, **285**(2): 182-189.
7) Schnell O, Hummel M, Weber C: **Economic and Clinical Aspects of Diabetes Regarding Self-Monitoring of Blood Glucose**. *Diabetes Technology and Therapeutics* 2008, **10** (supplement 1).
8) Garg, SK: **Glucose Monitoring: An Important Tool for Improving Glucose Control and Reducing Hypoglycemia**. *Diabetes Technology & Therapeutics* 2008, **10**(s1): S-1-S-4.
9) Naik RG, Ellis SL: **Self-Monitoring of Blood Glucose in Insulin-Requiring Type 2 Diabetes**. *Diabetes Technology & Therapeutics* June 2008, **10**(s1): S-67-S-71.
10) Juvenile Diabetes Research Foundation Continuous Glucose Monitoring Study Group: **Continuous glucose monitoring and intensive treatment of type 1 diabetes**. *New England Journal of Medicine* 2008, **359**(14): 1464-76.
11) Bailey TS, Zisser HC, Garg SK: **Reduction in hemoglobin A1C with real-time continuous glucose monitoring: results from a 12-week observational study**. *Diabetes Technology & Therapeutics* 2007, **9**(3): 203-10.
12) Deiss D, Bolinder J, Riveline JP, Battelino T, Bosi E, Tubiana-Rufi N, Kerr D, Phillip M: **Improved glycemic control in poorly controlled patients with type 1 diabetes using real-time continuous glucose monitoring**. *Diabetes Care* 2006, **29**(12): 2730-2.
13) The Juvenile Diabetes Research Foundation Continuous Glucose Monitoring Study Group: **The Cost-Effectiveness of Continuous Glucose Monitoring in Type 1 Diabetes**. *Diabetes Care* 2010, **33**(6):1269-74.
14) Palmer AJ, Roze S, Valentine WJ, Minshall ME, Foos V, Lurati FM, Lammert M, Spinas GA: **Validation of the CORE Diabetes Model against epidemiological and clinical studies**. *Current Medical Research & Opinion* 2004, **20** Suppl 1:S27-40.
15) Palmer AJ, Roze S, Valentine WJ, Minshall ME, Foos V, Lurati FM, Lammert M, Spinas GA: **The CORE Diabetes Model: Projecting long-term clinical outcomes, costs and cost-effectiveness of interventions in diabetes mellitus (types 1 and 2) to support clinical and reimbursement decision-making**. *Current Medical Research & Opinion* 2004, **20** Suppl 1:S5-26.
16) The C.D.C. Cost-Effectiveness Group: **Cost-effectiveness of Intensive Glycemic Control, Intensified Hypertension Control, and Serum Cholesterol Level Reduction for Type 2 Diabetes**. *JAMA* 2002, **287**(19): 2542-2551.

17) Hoerger TJ, Harris R, Hicks KA, Donahue K, Sorensen S, Engelgau M: **Screening for type 2 diabetes mellitus: a cost-effectiveness analysis**. *Annals of Internal Medicine* 2004, **140**(9): 689-99.
18) Klein BE, Klein R, McBride PE, Cruickshanks KJ, Palta M, Knudtson MD, Moss SE, Reinke JO:. **Cardiovascular Disease, Mortality, and Retinal Microvascular Characteristics in Type 1 Diabetes: Wisconsin Epidemiologic Study of Diabetic Retinopathy**. *Archives of Internal Medicine* 2004, **164**(17): 1917-1924.
19) Wu SY, Sainfort F, Tomar, RH, Tollios JL, Fryback DG, Klein R, Klein BE: **Development and application of a model to estimate the impact of type 1 diabetes on health-related quality of life**, *Diabetes Care* 1998, **21**(5): 725-731.
20) **The Absence of a Glycemic Threshold for the Development of Long-Term Complications: The Perspective of the Diabetes Control and Complications Trial:** *Diabetes* 1996, **45**(10): 1289-1298.
21) Selvin E, Marinopoulos S, Berkenblit G, Rami T, Brancati FL, Powe NR, Golden SH: **Meta-Analysis: Glycosylated Hemoglobin and Cardiovascular Disease in Diabetes Mellitus**. *Annals of Internal Medicine* 2004, **141**(6): 421-431.
22) Sullivan PW, Ghushchyan, V. **Preference-Based EQ-5D Index Scores for Chronic Conditions in the United States**. *Medical Decision Making* 2006, **26**: 410 - 420.
23) Gold M, Siegel JE, Russell LB, Weinstein MC: Cost-Effectiveness in Health and Medicine. New York: Oxford University Press; 1996.
24) **Diabetes Mall for Continuous Glucose Monitoring** [http://www.diabetesnet.com/diabetes_technology/continuous_monitoring.php]
25) Cusick M, Meleth AD, Agron E, Fisher MR, Reed GF, Knatterud GL, Barton FB, Davis MD, Ferris FL 3rd, Chew EY, Early Treatment Diabetic Retinopathy Study Research Group:**Associations of mortality and diabetes complications in patients with type 1 and type 2 diabetes: early treatment diabetic retinopathy study report no. 27**. *Diabetes Care* 2005, **28**(3): 617-25.
